# Enhancing teamwork in higher education: Experiences of podiatry students using SPARK^PLUS^ for self‐ and peer‐assessment in group work

**DOI:** 10.1002/jfa2.70010

**Published:** 2024-11-18

**Authors:** Michelle R. Kaminski, Anita Raspovic, Shannon E. Munteanu

**Affiliations:** ^1^ Discipline of Podiatry School of Allied Health, Human Services and Sport La Trobe University Melbourne Victoria Australia; ^2^ Department of Podiatry Monash Health Melbourne Victoria Australia; ^3^ School of Primary and Allied Health Care Monash University Melbourne Victoria Australia

**Keywords:** allied health students, assessment, group work, health education, podiatry

## Abstract

**Background:**

Teamwork is essential for delivering high‐quality healthcare, particularly given the increasing complexity of care due to chronic diseases, comorbidities and limited resources. The necessary skills and attributes for effective teamwork are often taught and assessed through group work within healthcare education programs. While group work can assist the development of skills and attributes of students to be effective team members, it also presents challenges, such as ensuring equitable student contributions. The Self and Peer Assessment Resource Kit (SPARK^PLUS^) endeavours to increase transparency, address inequities and provide learning‐oriented feedback in group work. However, there is limited evidence on its utility within university health education. This study was conducted with the aim of evaluating the experiences of podiatry students who used SPARK^PLUS^ for self‐evaluation and peer review during group work.

**Methods:**

Undergraduate podiatry students (*n* = 102) enrolled in a fourth‐year (final year) project‐based subject provided self‐ and peer‐assessment ratings for two group assessment tasks using SPARK^PLUS^. Eleven students (10.8%) participated in semi‐structured focus groups, which were audio recorded and transcribed verbatim. Inductive thematic analysis was conducted by two independent reviewers.

**Results:**

Four overarching themes and 11 subthemes were identified: (i) Performance (1a. Equity; 1b. Accountability), (ii) Peer inter‐relationships (2a. Social dynamics; 2b. Fear of consequences), (iii) Feedback and reflection (3a. Self‐reflection; 3b. Receiving and responding to feedback; 3c. Supervisor action; 3d. Avoiding confrontation) and (iv) Utility (4a. Enablers; 4b. Barriers; 4c. Integration throughout course).

**Conclusions:**

Overall, SPARK^PLUS^ encouraged equity, accountability and self‐reflection among group members. There was perceived risk of negative group dynamics and relationships when poor feedback was given. It was equivocal if team member contributions increased. Self‐ and peer‐assessment with SPARK^PLUS^ is a useful approach towards addressing inequities in group work within health education and may offer insight into the development and assessment of teamwork capabilities.

**Abbreviations**
RPF: relative performance factor
SA/PA: self‐assessment to peer‐assessment factor
SPARK^PLUS^
: the self and peer assessment resource kit

## 1 BACKGROUND

Teamwork is essential for the delivery of high‐quality healthcare, particularly due to the increasing complexity of care in the context of chronic disease, comorbidities and limited resources [1, 2]. Effective teamwork within healthcare settings has significant implications for patient safety, patient‐ and service‐related outcomes and the patient and staff experience [2, 3, 4, 5, 6, 7]. Therefore, it is critical that teamwork capabilities are developed and nurtured throughout a student's health education journey [2, 6]. High‐performing healthcare teams have been reported to share a number of characteristics including high levels of mutual trust and respect, shared purpose or goals, effective leadership, high‐level interpersonal skills, ability to problem solve collaboratively and appreciation for diversity [2, 4, 8, 9, 10]. While it is unclear *how* best to support healthcare students to develop these skills and attributes [1, 2, 6], there is a growing body of evidence to support the value of developing teamwork capabilities in health education [2, 6, 11, 12, 13, 14].

Teamwork is a critical capability for all healthcare workers from the medical, nursing and allied health sectors. The skills and attributes of teamwork are often taught and assessed through group work within undergraduate and postgraduate health programs. Building of teamwork capabilities has numerous potential advantages for health students including the ability to develop a shared identity, building of competence and self‐confidence and sharing of perspectives while challenging assumptions. It can also facilitate the development of communication and collaboration skills, such as the ability to give and receive constructive feedback and explore approaches to managing differences, all of which are important graduate attributes [6, 15, 16, 17, 18]. Within the podiatry discipline, and similarly to all other allied health professions, teamwork is one of the key capabilities for new graduates, as reflected in the Australian professional capabilities for podiatrists [19]. The podiatry curriculum is designed to satisfy accreditation needs [19] by delivering subjects that use group work and associated assessments as a vehicle to develop and assess students' capabilities related to teamwork.

Within tertiary education, learning activities associated with group work can support students to develop capabilities to be effective team members within healthcare, where teamwork is essential [1, 2, 20]. Although group work offers significant benefits, it also poses challenges. A major issue for educators when designing curricula using group‐based projects is to ensure equitable student contribution which is validly reflected in marks. This is also a primary concern of students, and if lacking, student motivation and satisfaction, particularly for higher‐performing students, can decline [21]. In turn, students have reported that group projects can feel unfair and demotivating [22], particularly if they deem their success depends on other team members who do not participate to the same level. Assessing individual students' learning performance and contribution as part of a group could address these concerns [16, 21, 22].

Self‐ and peer‐assessment is one method that can potentially address some of the inequities of group work [23, 24, 25, 26, 27, 28, 29] while also assisting in developing teamwork skills through the promotion of active rather than passive learning [6, 21]. While paper‐based self‐ and peer‐assessment tools are generally effective instruments for promoting equitable contributions in group work [28, 30, 31], they can be a burden to administer and lack student confidentiality. The development of online systems can overcome these problems with an Australian‐based system, the Self and Peer Assessment Resource Kit (SPARK^PLUS®^), showing much potential. To date, SPARK^PLUS^ has been implemented at 53 universities internationally [22].

SPARK^PLUS^ is an online (or digital) assessment tool that aims to increase group work transparency, address inequities and provide learner‐oriented feedback to complete the learning cycle [22, 27]. The tool allows students to confidentially rate their own and their peers' contributions to a team activity or project. Shared team marks can then be moderated based on individual contributions; the automation feature of the tool collates the data, calculates individual and peer contribution scores and distributes feedback and results to users. Other features of the tool include its user‐friendly setup and functionality, and being cloud‐based, this eliminates the need for server access [22, 27].

Previous studies have shown that SPARK^PLUS^ can be used to develop professional skills, provide timely feedback to large student cohorts, address inequities in group work and improve team dynamics and engagement [24, 27, 29]. While the use of SPARK^PLUS^ for self‐ and peer‐assessment has previously been evaluated in higher education within the fields of engineering and accounting [23, 24, 25, 26, 27, 29, 32, 33], there is limited evidence for its use in health education. Therefore, it is unclear whether findings from previous studies can be generalised to health students. Exploring group work initiatives through self‐ and peer‐evaluation may address the challenges of developing and assessing teamwork capabilities, aligning with the necessity of fostering effective teamwork in healthcare education. This approach is likely to ensure that students not only acquire essential skills but are also fairly evaluated for their contributions to group work. Ultimately, this may support the overarching goal of producing competent, collaborative healthcare professionals [34]. Therefore, the aim of this study was to evaluate podiatry student experiences of using SPARK^PLUS^ for self‐ and peer‐assessment in group work.

## 2 METHODS

### 2.1 Participants

All students enrolled in the fourth‐year (final year) undergraduate podiatry project‐based subject in 2018 at La Trobe University in Melbourne, Australia (*n* = 102) were invited to participate in the semi‐structured focus groups.

### 2.2 Materials

This study is reported in accordance with the consolidated criteria for reporting qualitative research (COREQ) framework [35].

A semi‐structured focus group protocol was used to gain an in‐depth understanding of podiatry student experiences of using the SPARK^PLUS^ tool for self‐ and peer‐assessment in group work. We used a pre‐defined discussion guide (Supporting Information S1) for the semi‐structured focus groups to provide consistency in our approach [36]. The focus groups consisted of open‐ended questions to encourage open dialogue, reflection, sharing of perspectives and a flow of ideas among the groups. To gain relevant data to the research aims, questions were arranged into the following topics: (i) likes and dislikes, (ii) effect on group member contributions, (iii) suggested changes, and (iv) future use. Focus groups were audio recorded and then transcribed verbatim by an independent party (Way With Words Limited^©^). An independent reviewer thoroughly checked transcripts for accuracy on a word‐for‐word basis and amended as necessary. Field notes were not taken during or after the focus groups. Participants were not offered, nor did they receive any remuneration or compensation for their participation in this study. To ensure the anonymity of the study participants, pseudonyms have been used in this report.

### 2.3 Procedures

Ethical approval was obtained by the La Trobe University Human Research and Ethics Committee (HEC18294) and all participants gave written informed consent prior to data collection.

All students enrolled in the undergraduate podiatry project‐based subject in 2018 at La Trobe University in Melbourne, Australia (*n* = 102) were required to perform self‐ and peer‐assessments using SPARK^PLUS^ for two group assessment tasks: a 6000‐word group written project report and a 16‐min team oral presentation. The team for each assessment task consisted of five students. Students were responsible for their group allocations. The purpose of the group assessment tasks was to address several key learning outcomes. First, students were expected to critically reflect on various models of continuous quality improvement and their application within podiatry practice, integrating these models into the healthcare system. Second, students were to collaboratively apply project management principles to design and implement a quality improvement project, working within a specified timeframe. This involved not only executing and evaluating the project but also critically assessing its effectiveness and sustainability and generating recommendations for future improvements. Finally, students were required to effectively communicate the processes and outcomes of the project to a range of stakeholders, ensuring comprehensive dissemination and feedback. At the conclusion of the podiatry project‐based subject, all students received an email invitation to participate in the semi‐structured focus groups. Students were advised to contact the research team if they were interested in participating. Once participants expressed interest in participating, a face‐to‐face focus group date and time was provided.

Students were notified that SPARK^PLUS^ would be used for self‐ and peer‐assessment at the start of the semester for the project‐based subject via the following methods: (i) during their orientation session, (ii) via an announcement on the subject's Learning Management System homepage and (iii) via an email sent to students enrolled in the subject. Staff who taught into the podiatry project‐based subject were notified that SPARK^PLUS^ would be used at the start of the semester via email and had a briefing with the subject coordinator. In addition, students and staff were provided with written information and video instructions on how to complete the SPARK^PLUS^ tool.

Students within each group used SPARK^PLUS^ to rate each other and themselves according to six domains: (i) understanding what is required; (ii) level of enthusiasm and participation; (iii) suggesting ideas; (iv) performing tasks efficiently; (v) organising the team and ensuring things get done; and (vi) helping the group to function well as a team. For each domain, students used the following rating system: WB: Well Below Average; BA: Below Average; AV: Average; AA: Above Average; and WA: Well Above Average. Students also had the option to write a short comment regarding the performance of their team members for the task [22].

After taking into account all group members' ratings, SPARK^PLUS^ automatically calculated two assessment factors of interest: a Relative Performance Factor (RPF) and a Self‐Assessment to Peer‐Assessment (SA/PA) factor [22, 24, 29]. The RPF is determined using both the self‐ and peer‐assessment ratings of a student's contribution. This factor indicates a student's overall relative performance compared to their group members. A RPF value greater than one indicates that the student's performance is better than the average performance of their peers. Inversely, a RPF value less than one indicates the opposite. The RPF assessment factor was used to moderate individual marks based on their level of contribution for each of the two group assessment tasks using the following formula [22, 24, 29]:

Individualmark=TeammarkxIndividual’sRPF

The SA/PA factor is the ratio of a student's own rating of themselves compared to the average rating of their contribution by their peers. The SA/PA factor is particularly useful for providing strong feedback to students on their individual performance within the group. A SA/PA factor greater than one indicates that the student rated their own performance higher than the average rating they received from their peers and vice versa [22, 24, 29].

Two semi‐structured focus groups were conducted on November 26, 2018 in the Health Sciences Clinic at La Trobe University, Melbourne, Australia to explore the podiatry students' experiences of using SPARK^PLUS^ for self‐ and peer‐assessment in group work. The average duration of the focus groups was 26.4 min. Our research team included researchers with experience in quantitative and qualitative research investigating foot and ankle pathology, chronic disease, psychology and health education. The semi‐structured focus groups were facilitated by a Lecturer in Podiatry (MRK, PhD, female identifying) with five years' experience and an Associate Professor of Podiatry/Course Coordinator (AR, PhD, female identifying) with 20 plus years' experience in higher education. The thematic analysis was conducted by the same Lecturer (MRK) and a Professor of Podiatry/Discipline Lead (SEM, PhD, male identifying) with 20 years' experience in higher education.

The trustworthiness of the study findings, which encompasses credibility, dependability and transferability of the data, was ensured through several approaches [37, 38]. To ensure credibility, all students (*n* = 102) within the podiatry project‐based subject were invited to participate. For those students that volunteered (*n* = 11), we conducted two separate focus groups in which the participants were found to have a range of individual RPF values within the sample. This may have therefore contributed to a richer variation of the factors under study [38]. To ensure dependability, focus groups were audio recorded and inductive thematic analysis was conducted systematically by two independent reviewers with diverse clinical and research backgrounds. In addition, the two reviewers met regularly to discuss coding, candidate themes and subthemes to ensure the analysis maintained rigour and consistency [38, 39]. Transferability of the study was supported by giving a clear and distinct description of the study context, outlining the recruitment and characteristics of participants and providing transparent reporting of the data collection and statistical analysis processes [38].

### 2.4 Data analysis

Inductive thematic analysis was applied by two independent reviewers following the framework outlined by Braun and Clarke (2006) [40]. This was to ensure both a systematic and objective process. Initially, the two independent reviewers (MRK and SEM) immersed themselves within the focus group transcripts (Phase 1); the first reviewer (MRK) was not involved in the transcription process and the second reviewer (SEM) was not involved in the focus group process, nor the transcription process. Open coding of each transcript was manually undertaken by two independent reviewers (MRK and SEM) on hard copies of the entire dataset (Phase 2). Codes from both reviewers were then collated and clustered using a secure web‐based list‐making software program (Trello^®^, Atlassian, New York, USA) into candidate themes and subthemes by one reviewer (MRK) and were checked for accuracy by the second reviewer (SEM) (Phase 3). Themes were reviewed and validated against coded extracts and the entire dataset by two independent reviewers (MRK and SEM) (Phase 4). Final themes were agreed by the consensus of the two reviewers (MRK and SEM) and any disagreements were resolved by a third party (AR) (Phase 5). All authors (MRK, SEM and AR) were involved in the final analysis and selection of extracts for this publication (Phase 6) [40]. Although data were collected in November 2018, there were delays to data analysis caused by disruptions created by the COVID‐19 pandemic.

## 3 RESULTS

Two semi‐structured focus groups were conducted with 11 (10.8%) podiatry students. This included 6 females and 5 males. Participants were aged between 22 and 25 years. The first focus group (*n* = 6; 4 females, 2 males) had one student with a RPF < 1 and SA/PA > 1, three students with a RPF and SA/PA score of 1, one student with a RPF and SA/PA > 1 and one student with a RPF > 1 and SA/PA < 1, while the second focus group (*n* = 5; 2 females, 3 males) had one student with a RPF and SA/PA < 1, one student with a RPF < 1 and SA/PA > 1, two students with a RPF and SA/PA score of 1, one student with a RPF > 1 and SA/PA < 1. Table 1 presents the participant characteristics.

**TABLE 1:** Participant characteristics.

Pseudonym	Sex	Focus group	Project report		Team presentation	
			RPF	SA/PA	RPF	SA/PA
Jonathan	Male	1	1.00	1.00	1.00	1.00
Anna	Female	1	1.00	1.00	1.00	1.00
Jessica	Female	1	1.00	1.00	1.00	1.00
Steven	Male	1	0.97	1.10	0.98	1.09
Rachel	Female	1	1.03	1.28	1.02	1.22
Amy	Female	1	1.10	0.95	1.04	0.99
Theodore	Male	2	1.00	1.00	1.00	1.00
Claire	Female	2	1.00	1.00	1.00	1.00
Paul	Male	2	0.95	1.04	0.99	1.02
Larry	Male	2	0.99	0.87	0.99	0.87
Leanne	Female	2	1.01	0.95	1.01	0.88

*Note*: RPF, Relative Performance Factor. SA/PA, Self‐Assessment to Peer‐Assessment factor. RPF < 1 (performance is less than the average performance of their peers); RPF = 1 (performance is equal to their peers); RPF > 1 (performance is more than the average performance of their peers). SA/PA < 1 (self‐performance rating is less than the average performance rating from peers); SA/PA = 1 (self‐performance rating is equal to the average performance rating from peers); SA/PA > 1 (self‐performance rating is more than the average performance rating from peers).

**FIGURE 1:**
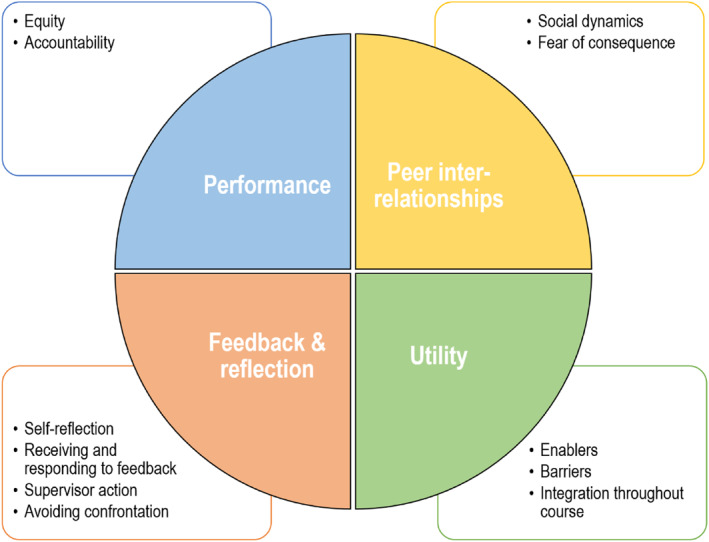
Thematic analysis mind map.

The themes presented represent contributions from podiatry students with a primary focus on their experiences of using the SPARK^PLUS^ tool for self‐ and peer‐assessment in group work. Four over‐arching themes and 11 subthemes (Figure 1) were identified: (i) Performance (1a. Equity; 1b. Accountability), (ii) Peer inter‐relationships (2a. Social dynamics; 2b. Fear of consequences), (iii) Feedback and reflection (3a. Self‐reflection; 3b. Receiving and responding to feedback; 3c. Supervisor action; 3d. Avoiding confrontation) and (iv) Utility (4a. Enablers; 4b. Barriers; 4c. Integration throughout course).

### 3.1 Theme 1: Performance

#### 3.1.1 1a. Equity

Theme 1a provides insight into student perceptions of the efficacy of the SPARK^PLUS^ tool in addressing inequities in group work. Students reported that the SPARK^PLUS^ tool allowed for improvements in the fairness of mark allocations and equity among group members, as marks should be based on contribution to the group task. One student described the importance of providing fair marks to the high‐performing group members compared to the low‐performing group members:

“…it’s really important that the marks are given to the actual person who does most of the work. And, the people who don’t do any work should be, not punished, but…” (Rachel, RPF and SA/PA >1, focus group 1)

Several students reported feeling frustrated when low‐contributing group members received the same marks as high‐contributing group members and that it “*took quite a toll on the group*” (*Leanne*, *RPF* >*1 and SA/PA* <*1*, *focus group 2*). Students reflected on their frustrations of group work relating to fairness of marks:

“…we’ve had people who just haven’t done anything, and in the end we’ve just been, like, it’s just going to be easier for us to do it ourselves kind of thing. But then they still get your mark, and it’s a bit frustrating” (Claire, RPF and SA/PA = 1, focus group 2)

The students thought that the SPARK^PLUS^ tool may address this issue and help to reduce frustration among group members. One student expressed that their group members were relieved that there was now something available to document contributions:

“…they’re [group members] glad that there is something there now that allows them to say, um, the contributions…they were kind of frustrated about it [poor contributions of some group members]” (Leanne, RPF >1 and SA/PA <1, focus group 2)

Fairness of marks was deemed more important than negative responses associated with providing or receiving poor feedback, as one student advised:

“I feel bad [giving a low score to peer], but I feel like they deserve it… I can’t mark them good because they didn’t really do much work” (Rachel, RPF and SA/PA >1, focus group 1)

Students felt satisfied by being able to give recognition and/or praise to high‐performing group members. One student expressed:

“There was a guy in our group who did a huge amount of work, over and above what was required, and yes, it was just good to be able to reward him for it” (Steven, RPF <1 and SA/PA >1, focus group 1)

It was equivocal as to whether SPARK^PLUS^ increased team member contributions; the magnitude of contribution was also unclear. One student reflected that there was no change to contributions from low‐performing group members:

“I think the ones that didn’t do too much, continued not to do too much” (Amy, RPF >1 and SA/PA <1, focus group 1)

#### 3.1.2 1b. Accountability

Theme 1b illustrates student experiences and their perceptions of how the SPARK^PLUS^ tool made students accountable for their level of contribution. Students conveyed that their ability to report and document poor contribution was important to them:

“I think sometimes with groups you can have people who don’t do what they should and it’s always that awkward one of how do you approach saying that…” (Jonathan, RPF and SA/PA = 1, focus group 1)

Being held accountable for your own and group members' individual contributions and the associated influence on marks was also important. Two students reflected on their experiences and perceptions of accountability when using the SPARK^PLUS^ tool:

“…she’ll [poor performing group member] see that and think, oh, well, obviously I didn’t do as good a job as I thought I had. But that’s a good thing… that someone actually learns from that” (Amy, RPF >1 and SA/PA <1, focus group 1)

“I feel like because everyone knows they’re getting graded they’re going to put in for it, because they know other people have an opinion on how much they’re doing. So, it kind of gives them that push to be like, okay, well now I really have to pull my weight and, yeah, do what’s necessary, or else, yeah, they can, I guess, report it.” (Leanne, RPF >1 and SA/PA <1, focus group 2)

### 3.2 Theme 2: Peer inter‐relationships

#### 3.2.1 2a. Social dynamics

Theme 2a addresses the impact that the SPARK^PLUS^ tool had on social dynamics and relationships within the group. Some students expressed that providing poor feedback to their peers may negatively affect the social dynamics and relationships among group members. For example, cause tension between group members and risk adversely affecting student relationships. One student reflected on their own negative experience of being involved in group work:

“After it’s done [group work], it crushes all friendships… group work can be really hard” (Rachel, RPF and SA/PA >1, focus group 1)

Two students reflected on the potential impact of giving poor feedback on group dynamics and relationships:

“All of a sudden now you’ve sort of just put a whole lot of tension inside the group and now you’re expected to do the next eight weeks together” (Jonathan, RPF and SA/PA = 1, focus group 1)

“If you start using it [SPARK^PLUS^] in second and third year, you could get yourself offside with people [group members] and then by the fourth year, no one will work with you” (Amy, RPF >1 and SA/PA <1, focus group 1)

Students conveyed that contribution is positively influenced by the freedom to choose group members and good group dynamics. Students thought that the SPARK^PLUS^ tool may be more effective in increasing contributions in groups with poor dynamics.

#### 3.2.2 2b. Fear of consequences

Theme 2b provides insight into student perceptions of providing poor feedback to their peers, specifically outlining the fear of consequences and associated guilt. Students generally lacked courage to provide poor feedback to peers due to fear of confrontation. For example, feeling uncomfortable with being accountable for feedback and how the person would respond:

“…I don’t want to mark people too low, ‘cause they might see what I’ve given them” (Leanne, RPF >1 and SA/PA <1, focus group 2)

Students expressed feelings of guilt associated with providing poor feedback and its effect on their peers' individual marks:

“…if you’re someone that might have been below average, but I didn’t really want to do it, because I didn’t want to affect their mark. Like I didn’t know how much it would affect their mark…‘cause they might have, you know, just been a little bit lower than everyone else, but if they get affected a lot then you didn’t really want to do it” (Larry, RPF and SA/PA <1, focus group 2)

“…you kind of feel a bit bad, if it was like, your friend, and it’s going to affect their mark” (Claire, RPF and SA/PA = 1, focus group 2)

Students had a conscience associated with giving poor feedback and the risk of upsetting peers. One student reflected on their conscience and feelings of guilt when using the SPARK^PLUS^ tool:

“…people in a group might think that they are contributing, and then if you’ve got that comment [poor feedback from peers]…you might be really upset about it” (Claire, RPF and SA/PA = 1, focus group 2)

### 3.3 Theme 3: Feedback and reflection

#### 3.3.1 3a. Self‐reflection

Theme 3a describes how students found the SPARK^PLUS^ tool useful in self‐reflective practices. Students thought that the SPARK^PLUS^ tool allowed for self‐reflection and provided insight into their own performance and whether adjustments were needed for their future behaviour. Two students described how the tool allowed for self‐reflection:

“…it kind of made you think, okay, I need to, you know, start picking my game up if I’m not doing as much as everybody else thought I was doing. Or, I’m taking too much of it and everybody else is thinking maybe I’m doing too much… taking over the whole group assignment and nobody else doing anything…” (Anna, RPF and SA/PA = 1, focus group 1)

“…it would actually like be able to teach people for the future to improve on what they’ve done wrong or what they’ve done right to continue doing that” (Steven, RPF <1 and SA/PA >1, focus group 1)

#### 3.3.2 3b. Receiving and responding to feedback

Theme 3b explores student experiences in receiving and responding to peer feedback. Students believed that given there is variability in the way people respond to feedback, group dynamics and peer relationships may be negatively affected when using the tool. One student reflected that this may be related to the individual's response or the group dynamics:

“I think it also depends on the way that certain people would take certain feedback. Because, you know, everyone obviously responds…different ways to things” (Jonathan, RPF and SA/PA = 1, focus group 1)

“…but, of course, that comes down to group dynamics, I guess. How people cop criticism…” (Jonathan, RPF and SA/PA = 1, focus group 1)

#### 3.3.3 3c. Supervisor action

Theme 3c describes student perceptions on the importance of supervisor action and involvement in cases of poor‐performing group members. Students thought providing feedback was important as it allows the supervisor to address poor performance and assist with group issues. For those in groups with poor dynamics, one student reflected on the benefits of supervisor action and input to close the feedback loop:

“…for those who don’t have that, don’t have a well‐connected group, you can report or say that you’re concerned through this program [to the supervisor]. So, I felt that extra focus was really nice” (Theodore, RPF and SA/PA = 1, focus group 2)

#### 3.3.4 3d. Avoiding confrontation

Theme 3d illustrates student experiences of using the SPARK^PLUS^ tool to avoid confrontation when faced with poor‐performing group members. As to avoid confrontation, students liked that the SPARK^PLUS^ tool allowed for negative feedback to be shared between group members rather than from the individual. Two students reflected on their experiences and perceptions of avoiding confrontation when providing peer feedback:

“I’m not someone who likes confrontation, so I was thinking this person’s really annoying me, they’re not lifting their weight, but hang on, I’ve got SPARK^PLUS^, I’ll worry about that later. I can just mark them down then” (Amy, RPF >1 and SA/PA <1, focus group 1)

“…it’s pretty easy to hide behind a computer screen though” (Steven, RPF <1 and SA/PA >1, focus group 1)

### 3.4 Theme 4: Utility

#### 3.4.1 4a. Enablers

Theme 4a discusses the benefits and enablers of the SPARK^PLUS^ tool for self‐ and peer‐assessment in group work. Students thought that the SPARK^PLUS^ tool provided a confidential and convenient platform to provide feedback and communication to peers and supervisors. Students advised that the tool was quick and easy to use and that it was superior to paper‐based peer feedback and assessment forms. Four students commented on the utility of the tool:

“…really quick to do. It took less than five minutes to do it, which was really good” (Larry, RPF and SA/PA <1, focus group 2)

“…it’s a helpful tool in terms of providing feedback for everyone in the team. As well as it’s very easy to navigate around. And with the feedback section it’s really good for providing constructive feedback and areas to commend everyone’s effort” (Paul, RPF <1 and SA/PA >1, focus group 2)

“Gave you a platform where you could say anonymously” (Jonathan, RPF and SA/PA = 1, focus group 1)

“It wasn’t hard to navigate through the questions and to use the rating system…very easy for anyone to use” (Amy, RPF >1 and SA/PA <1, focus group 1)

#### 3.4.2 4b. Barriers

Theme 4b considers the barriers of using the SPARK^PLUS^ tool for self‐ and peer‐assessment in group work. While the use of SPARK^PLUS^ was positively viewed by the students, there were several barriers identified. Students commented that the tool assumes all group members are working to the same schedule, which therefore forces pre‐allocation of project tasks. This may not be feasible for all group members nor the optimal method for completing the project. For example, semester‐long project‐based subject with students participating in clinical placements in other subjects that they are concurrently enrolled in. Some students were frustrated that there was a delay in the release of marks due to some students not completing the tool. In addition, automated reminders to complete the tool were sent out to all students, despite many having completed it.

Students thought that being unfamiliar with the workings of the system may have reduced the effectiveness of the tool and the completion of all components:

“…because we weren’t really familiar with it, like we weren’t introduced earlier on, like maybe, third year or second year, to our group project, we might improve in terms of the effectiveness of the tool [if tool was introduced earlier in the course]” (Theodore, RPF and SA/PA = 1, focus group 2)

For some students, the automation of the scores was confusing and students perceived that this hindered their ability to score peers more or less:

“…at the start I wasn’t really sure that when you clicked average it just meant that everyone contributed equally… and it was kind of a bit confusing cause you were kind of going, do I click above average for people who did more, and then less for people who did less?” (Leanne, RPF >1 and SA/PA <1, focus group 2)

The students thought the automation feature and the completion of the mandatory fields were unclear and recommended that educators should provide further explanation and support to students regarding this. It was apparent that some students lacked the skills and clarity on how to be an effective and well‐functioning group member; therefore, students were unclear on how to provide appropriate ratings and feedback to group members:

“I don’t think I even left any comments, ‘cause I kind of didn’t know what to write” (Claire, RPF and SA/PA = 1, focus group 2)

Some students were concerned that individuals who were contributing above what was required may be over‐rewarded, and inversely those who were completing the expected workload may have been penalised.

#### 3.4.3 4c. Integration throughout course

Theme 4c explores student insights on how the SPARK^PLUS^ tool could be integrated throughout the podiatry course. Students recommended for the tool to be used earlier in the course or cross‐disciplines for improving familiarisation of the tool, addressing bad habits and promoting effective group work and individual contributions; particularly for heavily weighted subjects. Several students provided suggestions for the future integration of the SPARK^PLUS^ tool in the podiatry course:

“…by using earlier as well, people become more familiar with the tool, so it just makes it easier” (Theodore, RPF and SA/PA = 1, focus group 2)

“So, they [poor performing group members] don’t get to fourth year still with some bad habits” (Amy, RPF >1 and SA/PA <1, focus group 1)

“And I think if it’s done, like, start of second year or whatever, the people that don’t usually do the work might learn earlier, if they’ve got to learn by third year and fourth year, they’re contributing more. Like, ‘cause they’ve started on the process, I guess” (Larry, RPF and SA/PA <1, focus group 2)

## 4 DISCUSSION

This study has addressed an important gap in the existing health education literature by providing insights into undergraduate student experiences of self‐ and peer‐assessment using an online, cloud‐based assessment tool. Overall, our findings demonstrate that self‐ and peer‐assessment using SPARK^PLUS^ encourages equity, accountability and self‐reflection among group members, yet there is perceived risk for negative effects on group dynamics and relationships when poor feedback is given. More specifically, our study found that self‐ and peer‐assessment benefited students by the promotion of more equitable assessment and fairness of marks, encouraged accountably of group member contributions, allowed self‐reflection and insight into their own performance and provided a confidential and convenient platform to provide feedback to peers and supervisors. These findings are consistent with other studies that have evaluated the use of SPARK^PLUS^ in the fields of engineering and accounting [23, 24, 25, 26, 27, 29, 32, 33].

While SPARK^PLUS^ has previously been shown to facilitate confidential self‐ and peer‐assessment and encourage engagement, learning and the development of new skills required for teamwork [24, 25, 27, 29], a point of difference in our study was that it was equivocal as to whether it increased team member contributions. One explanation for this is that a high proportion of students in our sample (72.7%) were classified as higher‐performing students (i.e. with a RPF score of one or above). These students may have experienced better group dynamics, leading to more equal contributions from team members. However, one study [23] has shown that this is not always the case.

Student concerns regarding self‐ and peer‐assessment were mostly related to the impact of poor feedback on group dynamics and relationships. Students also experienced feelings of guilt and fear of confrontation when having to provide poor feedback to their peers, which is consistent with a previous study [29] and health education literature [6]. Many of the barriers and negative experiences students faced with using SPARK^PLUS^ aligned with the capabilities required of them to be effective team members (e.g. guilt providing negative feedback). As such, the findings highlight the value in using regular peer‐assessment and student reflections as a means of improving student capabilities related to effective team membership. Encouraging students to provide feedback can lead to improvement in judgement, assessment ability and critical evaluation skills [29], all of which allows students to take more responsibility for their own learning. The need for developing capabilities in group work dynamics seems to be a common problem among undergraduate students [25, 29, 32]. Providing education to students on how to best deliver constructive feedback to peers, how to respond to and learn from constructive feedback and how to resolve group conflict is an important initial strategy [25, 29, 32, 41]. Students should be educated on the importance of timely and focused feedback and its potential to shape future learning and achievement [29]. The reluctance of students to provide constructive feedback to their peers, particularly to poor‐performing group members, also needs to be addressed [29]. While it is appropriate for students to look for guidance from educators or supervisors, there should also be some boundaries or negotiations set. It is also important that educators act on poor feedback by following up with poor‐performing students and the other group members, thereby, closing the feedback loop within the team.

The key components for implementing peer assessment activities within health education have been recently explored [6, 41]. It appears that for greater success and uptake, it may be important for educators to:

- Explain the purpose, aim and learning objectives of the peer evaluation;
- Explain the purpose of teamwork and set expectations;
- Orientate students to the peer assessment tool and provide supporting materials for its use;
- Ensure the same peer assessment tool is used throughout the curriculum;
- Ensure students are prepared and trained to give and receive constructive feedback;
- Develop positive relationships with students and foster a positive culture within the learning environment that is student‐centred;
- Encourage respect among students for the learning activity and for the collaboration of the team;
- Emphasise to students the importance of being self‐aware, well prepared and accepting responsibility for the peer assessment task;
- Assess teamwork over time;
- Provide student feedback in a professional and appropriate way;
- Maintain the integrity of peer assessments by using anonymity; and
- Be transparent and clear with students in how peer assessments will be used for individual grades [6, 41].

One of the core barriers highlighted by students when using SPARK^PLUS^ was that they were unclear on the workings of the system, how to use the software and how to rate the contribution of others, which is consistent with previous studies [27, 32]. Therefore, other strategies for improvement may include improving familiarisation of the tool by using it earlier in the course or across health disciplines and improving the effectiveness of the tool through building knowledge of its utility to both students and educators. For example, educating students on the many purposes of the tool, how scores are derived with the automation feature, how to complete mandatory fields and how to provide appropriate ratings based on student performance.

Our findings must be viewed considering some limitations. First, this study recruited participants from the podiatry discipline only; therefore, the findings may not be generalisable to other health disciplines. Second, the members of the research team involved in the focus groups were lecturers in the Discipline of Podiatry, which may have discouraged participants from criticising the tool. Although, this does not appear to be an issue in this study, as participants expressed both positive and negative experiences of the tool. Third, there was a higher proportion (72.7%) of students with a RPF score of one or above; therefore, the study sample may be more reflective of the experiences of higher‐performing students with potentially better group dynamics. This may explain why it was difficult to establish if the tool increased team member contributions or not. It is also likely that students with a RPF score of less than one (underperforming) would be in most need of SPARK^PLUS^ to maintain their engagement. Fourth, the data for this study were only collected from one subject over the course of one semester and specifically only included fourth‐year podiatry students. Therefore, it is unknown whether the student experience would have differed across year levels or during different time periods. Fifth, while our sample size of 11 participants is consistent with typical qualitative studies [36, 40, 42, 43], including more participants may have provided further insights and allowed us to reach data saturation. However, the concept of data saturation is often poorly defined and is difficult to assess in inductive thematic analysis [44, 45]. Overall, our study included a range of low‐ to high‐performing students, which allowed us to obtain detailed and relevant information on student experiences of self‐ and peer‐assessment. Sixth, we did not explore the ethnicity of participants; podiatry students were from a small university cohort (*n* = 102). Hence, we were not able to explore whether differing ethnicities and cultures influenced the student experience and their perceptions. Finally, we did not take field notes during or after the focus groups. Field notes may have enhanced the data and provided additional context for analysis, possibly leading to richer or additional themes.

## 5 CONCLUSIONS

Within the context of tertiary education, group work embedded within learning activities can foster the development of crucial skills needed for podiatry students to become effective team members in healthcare settings. Utilising SPARK^PLUS^ for self‐ and peer‐assessment presents a viable strategy to mitigate disparities in group work and enhance students' abilities to function effectively within teams. In summary, SPARK^PLUS^ was well accepted and positively viewed among the podiatry students. Students perceived that SPARK^PLUS^ promoted more equitable assessment and fairness of marks, encouraged accountably of group member contributions, allowed self‐reflection and insight into own performance and provided a confidential and convenient platform to provide feedback to peers and supervisors. However, there is potential for further refinement in this approach. Concerns arose from students regarding the impact of poor feedback on group dynamics and relationships, feelings of guilt and fear of confrontation when providing poor feedback, and at times, students were unclear on the workings of the system. Students were equivocal if the tool increased team member contributions. Strategies for improvement may include providing education to students on how to deliver constructive feedback to peers, how to resolve group conflict, providing familiarisation of the tool early and improving knowledge on the utility of the tool.

## AUTHOR CONTRIBUTIONS

**Michelle R. Kaminski**: Conceptualisation; data curation; formal analysis; investigation; methodology; project administration; visualisation; writing—original draft preparation; writing—review and editing. **Anita Raspovic**: Conceptualisation; formal analysis; investigation; methodology; visualisation; writing—review and editing. **Shannon E. Munteanu**: Conceptualisation; data curation; formal analysis; funding acquisition; methodology; project administration; visualisation; writing—review and editing.

## CONFLICT OF INTEREST STATEMENT

The authors declare that they have no competing interests.

## ETHICS STATEMENT

This study was approved by the La Trobe University Human Research and Ethics Committee (HEC18294). Written informed consent was obtained from all participants before taking part.

## CONSENT FOR PUBLICATION

Not applicable.

## Supporting information

Supporting Information S1

## Data Availability

The datasets used and/or analysed in the current study are available from the corresponding author on reasonable request.
