# Supplementary material for: Enhancing teamwork in higher education: Experiences of podiatry students using SPARKPLUS for self‐ and peer‐assessment in group work
Source: J Foot Ankle Res. 2024 Nov 18;17(4):e70010. doi: 10.1002/jfa2.70010 (PMC11573923; doi:10.1002/jfa2.70010)
Supplement: Supplementary file 1 — Supporting Information S1 [file JFA2-17-e70010-s001.pdf]

Appendix 1. Focus group discussion guide

STUDENT FOCUS GROUP DISCUSSION GUIDE

FOCUS GROUP PURPOSE & FRAMEWORK:

- To find out how the participants (students) felt about the acceptability of using the peer assessment tool, including if there were any difficulties with using the tool, and to gauge if it made any perceived differences to the contribution that they or their student peers made to their group assessment tasks, and to identify if there were any better ways that the tool could be used in the future
- Use the following questions to guide your discussion
- Feel free to follow a participant's lead, and explore their thoughts as they arise

INITIAL STATEMENTS TO PARTICIPANTS:

- Let the participant know that you will be asking them questions about their experience with use of SPARK^{PLUS} in the Podiatry Project Subject (POD5PPJ)
- Remind the participant that you will be recording their answers, so that the researchers can listen to them later
- Reassure the participant that they can give whatever answers they like, and that they won't get into trouble for saying what they think

Topic area:	Question examples:
**Context questions & general thoughts**	Tell me about the SPARK^{PLUS} peer assessment tool<br>What did you like about the tool?<br>What were the things you didn't like about the tool?<br>• Prompts: web interface, time to complete, ease of use, any concerns regarding effects on team dynamics<br>Did the tool make any difference to the contribution to group assessment tasks? From themselves and from their peers!
**Use of SPARK^{PLUS} in the future**	What would you change about the peer assessment tool?<br>Would you change anything about the way the peer assessment tool was used in the podiatry project-based subject?<br>Would you like to see the peer assessment tool used again in the podiatry project-based subject or other subjects? Why? Why not?
